# Biomimetic approaches and materials in restorative and regenerative dentistry: review article

**DOI:** 10.1186/s12903-023-02808-3

**Published:** 2023-02-16

**Authors:** Lamia Singer, Ahmed Fouda, Christoph Bourauel

**Affiliations:** 1 Oral Technology, University Hospital Bonn, 53111 Bonn, North Rhine-Westphalia Germany; 2 Department of Orthodontics, University Hospital Bonn, 53111 Bonn, North Rhine-Westphalia Germany; 3 Department of Fixed Prosthodontics, Suez Canal University, Ismailia, Egypt

**Keywords:** Biomimetic dentistry, Restorative dentistry, Regenerative medicine, Dentine remineralisation

## Abstract

Biomimetics is a branch of science that explores the technical beauty of nature. The concept of biomimetics has been brilliantly applied in famous applications such as the design of the Eiffel Tower that has been inspired from the trabecular structure of bone. In dentistry, the purpose of using biomimetic concepts and protocols is to conserve tooth structure and vitality, increase the longevity of restorative dental treatments, and eliminate future retreatment cycles. Biomimetic dental materials are inherently biocompatible with excellent physico-chemical properties. They have been successfully applied in different dental fields with the advantages of enhanced strength, sealing, regenerative and antibacterial abilities. Moreover, many biomimetic materials were proven to overcome significant limitations of earlier available generation counterpart. Therefore, this review aims to spot the light on some recent developments in the emerging field of biomimetics especially in restorative and regenerative dentistry. Different approaches of restoration, remineralisation and regeneration of teeth are also discussed in this review. In addition, various biomimetic dental restorative materials and tissue engineering materials are discussed.

## Background

The term “Biomimetics” is derived from the Latin words “bios” which means life and “mimesis” which means to copy or mimic [1]. Biomimicry is an interdisciplinary field of mimicking nature’s ideal biological approaches and strategies using chemistry, physics, mathematics, and engineering concepts to develop novel synthetic materials and organs [2]. Among the popular examples of biomimetics are the swimsuits motivated by the dermal denticles covering shark’s skin, the needles that are typically inspired by mosquitos, and the shape of wind turbine blades modelled from the humpback whale ‘fins [3].

History of biomimetics goes back to the first and second century where evidence of crude dental implants was seen in roman and pre-Columbian cultures of central and South America. In 659 AD, the first use of dental amalgam was found written in the Chinese literature. The use of heart pacemaker, hip and knee joint started in the beginning and middle of twentieth century. In 1950, the American inventor Otto Schmitt coined the term biomimetics while in 1960, the subject of copying, imitating, and learning from biology was named Bionics by Jack Steele [3, 4]. The term biomimetic was officially listed for the first time in the Webster’s Dictionary in 1974. Although biomimetic history goes back to the first century, it did not become popularized among scientists and researchers except after publishing a ground breaking book about it by author Janine Benyus, 1997 namely ‘Biomimicry: Innovation Inspired by Nature.’ [5].

Bio-mimetic dentistry is the art and science of repairing damaged teeth with restorations that imitate the living tissues (e.g., enamel, dentin, bone, cementum, etc.) in terms of appearance, function, and strength [6]. The secondary biomimetic goal is to develop restorative materials that can restore the biomechanics of the natural tooth. The applicability of biomimetics has been greatly considered at molecular levels in terms of promoting wound healing and soft and hard-tissue regeneration [7]. At macro structural level, biomimetic preservation of biomechanical, structural, and aesthetic integrity of teeth can be achieved by various biomimetic restorative materials [8]. For this purpose, materials scientists ideally take natural teeth as a reference during the development of dental restorative materials.

**Fig. 1:**
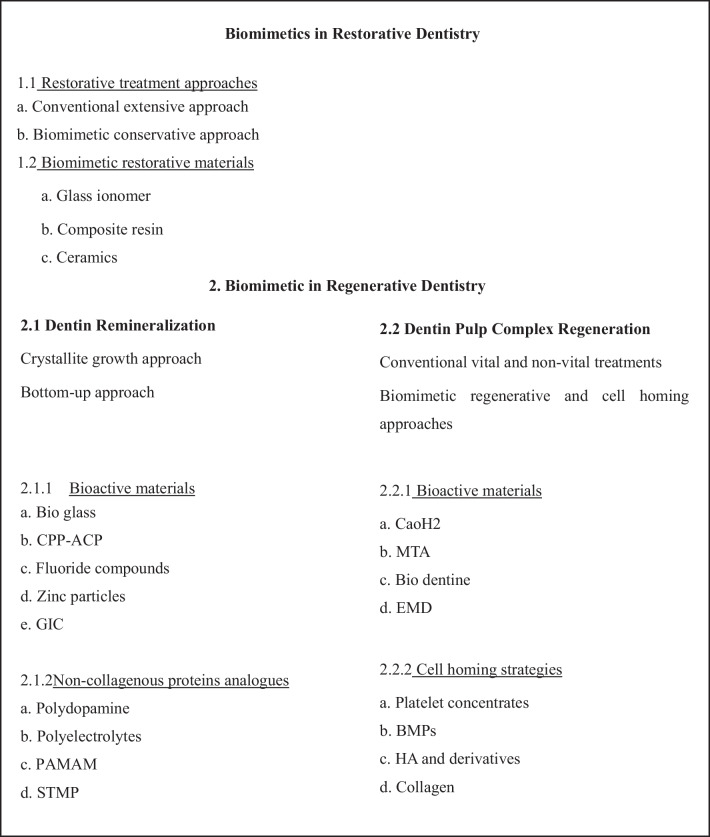
Scheme of emerging trends of biomimetic dentistry

The emerging trend of biomimetic approaches in dentistry have been employed for a range of applications, such as restoring tooth defects using bioinspired analogs to achieve remineralization, bioactive and biomimetic biomaterials, and tissue engineering for regeneration (Fig. 1) [8].

## Biomimetic restorative dentistry

### Restorative treatment approaches

The main objective of biomimetic restorative dentistry is to return the hard tissues (enamel, dentin, cementum) to attain full function through a restorative material that can mimic or restore the biomechanics of the natural tooth. This allows the tooth to function as one unit against functional forces and provides near normal biology, and aesthetics [2].

**Fig. 2:**
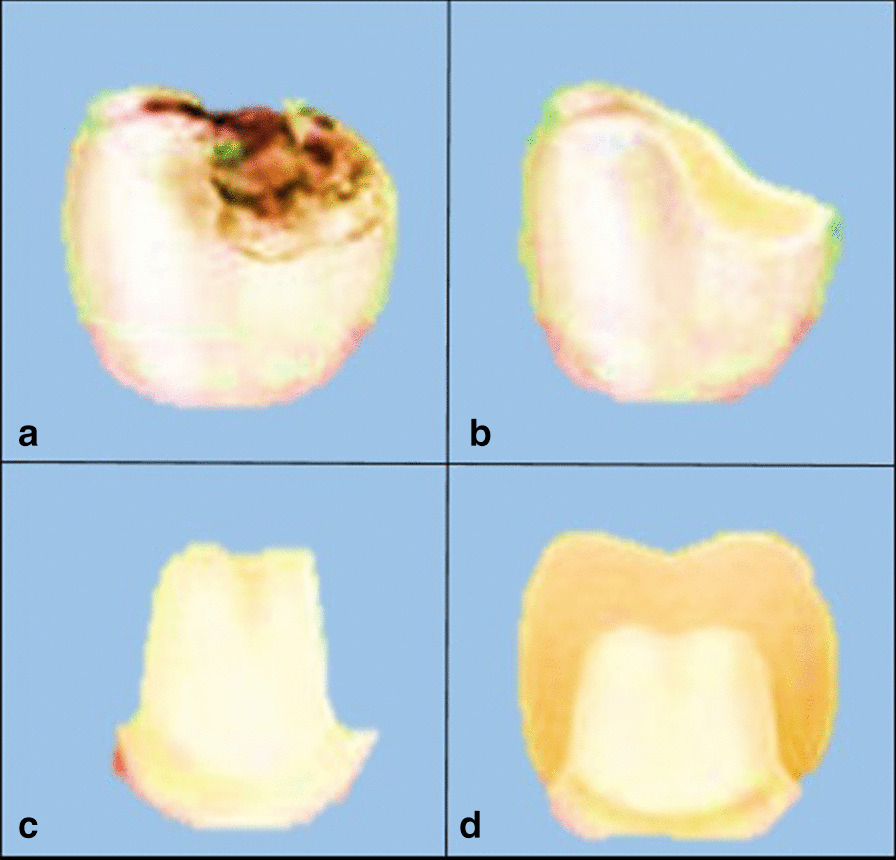
**a** Decayed tooth, **b** the decay is completely removed, **c** more tooth structure is removed to allow space for the placement of rigid restoration, **d** restoration is placed over the weakened tooth structure

**Fig. 3:**
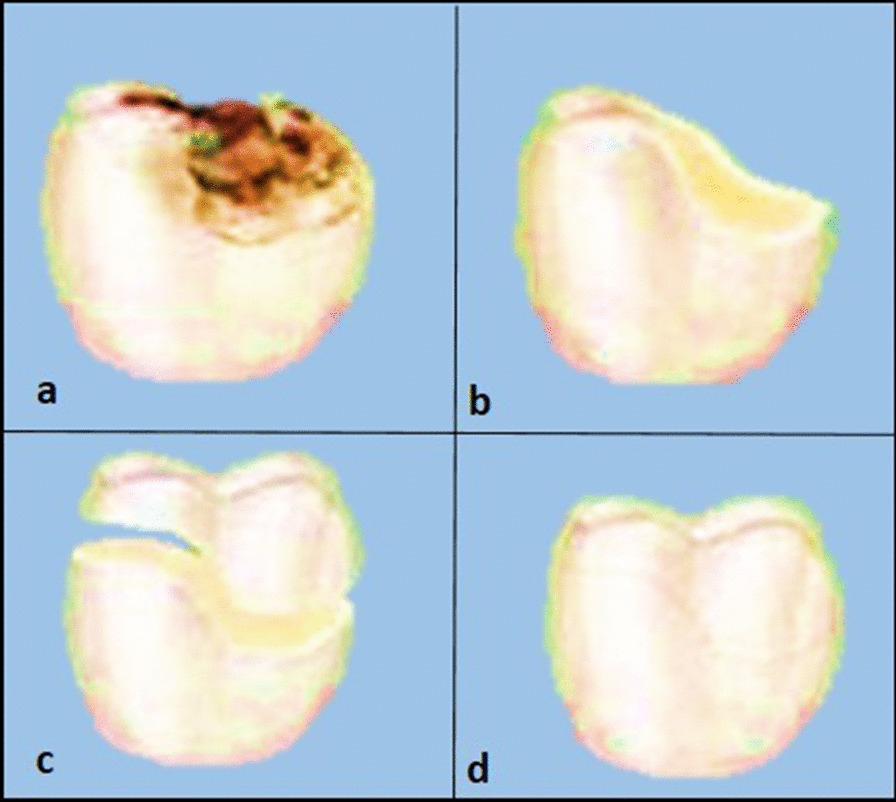
**a** Decayed tooth, **b** the decay is removed completely, **c** limited tooth structure is removed to maintain tooth strength, **d** restoration is bonded to the tooth restoring the structural integrity and strength

- *In conventional extension for prevention approach*, not only the diseased but also sound tooth structure are removed and replaced with rigid, non-responsive materials. This treatment plan usually weakens the remaining tooth structure and yields a short life span restoration (Fig. 2) [9].
- *In Biomimetic approach*, the concept of less or no dentistry is the best dentistry has been adopted. It is conservative and only focuses on restoring the teeth and simulating the natural dentition as much as possible. The biomimetic restorative protocols aim to achieve these results by stress-reducing protocols and bond-maximizing protocols. Cavities and other lesions are carefully repaired using advanced materials and adhesives so the tooth retains its inherent natural properties (Fig. 3) [9].

### Biomimetic restorative materials

- Glass ionomer (Man-made dentin)

Glass-ionomer (GIC) is the generic name of a group of materials that use silicate glass powder and aqueous solution of polyacrylic acid. They undergo a significant acid—base reaction as a part of their setting reaction and show a continuing fluoride release. Glass ionomer cements (GICs) are considered as biomimetic materials because they have the same coefficient of thermal expansion as that of tooth structure, bond adhesively to enamel and dentin, and release fluoride over a prolonged period of time [10].

GICs ability to release ions is responsible for the development of long-term durable bonds at the tooth/restoration interface. Studies have shown that ions can move from the cement and from the tooth into the interfacial region to create an ion-exchange layer that proved to be highly durable in clinical service [11]. In spite of these advantages, conventional glass ionomers suffer from disadvantages such as short working times, long setting times, brittleness, low fracture toughness, and susceptibility to moisture contamination or dehydration during the early stages of the setting reaction [12]. Various modifications in the powder and liquid of glass ionomer material have been done to improve its performance.

Nano-hydroxyapatite (HA) particles have been added to GIC to produce GIC-HA hybrids, which showed improved release of fluoride ions, and improved mechanical and antibacterial properties [13]. Moreover, Garoushi et al. [14] in a recent study explored the effect of addition of hollow and solid discontinuous glass fiber fillers on fracture toughness and flexural strength of GIC and resin modified GICs. Results showed significant enhancement in fracture toughness (280 and 200%) and flexural strength (170 and 140%) of hollow discontinuous glass fiber (10 wt %) added to GICs.

In 2020, Singer et al. [15] modified the antimicrobial activity of GIC with a mixture of three natural plant extracts* (Salvadora persica, Olea europaea*, and *Ficus carcia*). The three plant extracts improved the antimicrobial activity against *S. mutans* and against *M. luteus* while compressive strength was improved by addition of the plant extracts at higher concentrations.

b. Dental composite resin

Nearly everything in nature, including teeth, pearls, shells, corals, and bones, is composed of hybrid organic and inorganic composites with the structures of each component regulating the final performance of the hybrids [16]. Dental composite resin (DCR) represents an important category of hybrid biomaterials, which are composed of a resin matrix and inorganic fillers [17]. DCR has been widely applied in dentistry to restore diseased and defective teeth since the 1960s because of their excellent aesthetics, good biocompatibility, and ease of use [18].

Self-healing/Bleeding material is a resin composite system, which employs a biomimetic approach to perform a self-repairing function. Many natural materials are themselves self-healing composites as the natural bone, which has the ability to self-heal even with major fracture. Self-healing could be either intrinsic or extrinsic, according to whether the reparative molecules are produced only in response to damage (intrinsic) or are already deposited within the material (extrinsic) [19].

**Fig. 4:**
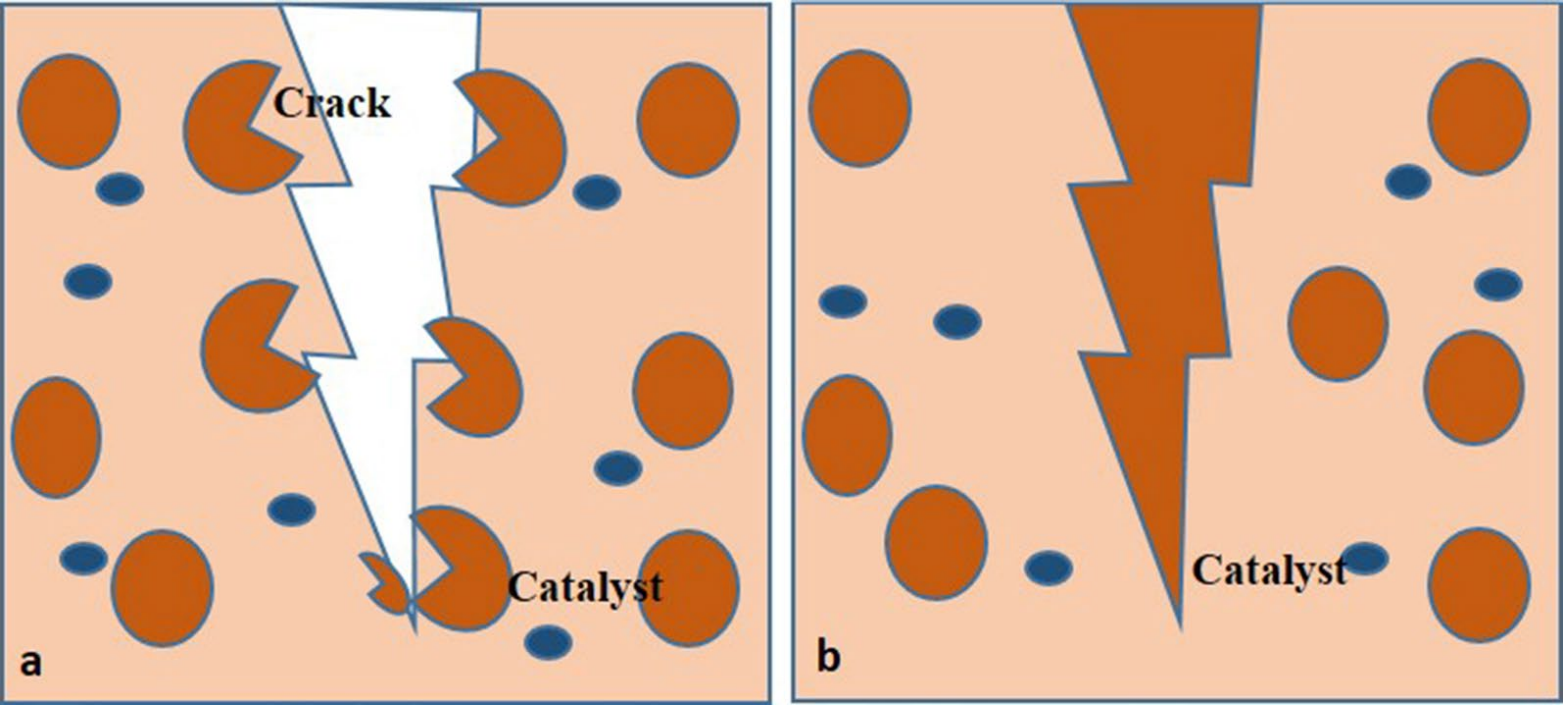
Self-healing composite resin **a** microcapsulesrupture and release of healing agents, **b** healing of the crack after polymerization of the healing agent by the catalyst

Intrinsic self-healing occurs at the molecular level, and an external source of energy is required to control the movement of the reactants [20]. On the other hand, extrinsic self-healing materials usually rely on polymeric capsules, which rupture near the crack and release the resin that reacts with an existing catalyst resulting in polymerization of the resin and crack repair (Fig. 4) [21, 22]. Visual enhancement of the damage could be achieved as well by the bleeding of a highly added conspicuous medium such as fluorescent dye [21, 22].

Bilayered resin composite structure is considered a new biomimetic restoration system, which mimics the fibrous structure of dentin-enamel complex [23]. It is made of a base formed of glass fiber reinforced composite resin (FRC) and a surface of a more polishable and wear resistant resin composite. The function of the FRC base for filling composites is to provide a crack propagation prevention layer for the restoration [24].

iii. Ceramics

Dental ceramics are able to mimic the natural appearance of the tooth. The popularity of dental ceramics has been widely extended in the last three decades after the evolution in computer based dental technologies and the introduction of the “digital workflow” concept in dentistry [25]. The ambition of researchers to develop biomimetic dental restorative ceramics existed long time ago. In 2000, Holland et al. were able to develop an apatite-leucite glass ceramics that are made of needle-like apatite building blocks similar to those found in the living dental tissues. The needle-like apatite crystals improved the esthetic and mechanical properties of the material [26–28].

Biomimetic dental ceramics should be able to establish gap free adhesion to the restored dental substance and to promote natural regeneration of surrounding tissues. Goudouri et al. [29] succeeded to incorporate an apatite-forming ability to a commercial dental restorative ceramic material to enhance the tissue attachment. The specimens demonstrated the formation of apatite-like layer on the surface without affecting the flexural strength of the material [30]. Biomimetic application of dental ceramics also includes bioactive coated ceramic implant. Several bioactive glass–ceramics are commercially available and were used to coat titanium and zirconia dental implants. The coating enhanced the osseo integration and tissue bonding around ceramic implants [31].

**Fig. 5:**
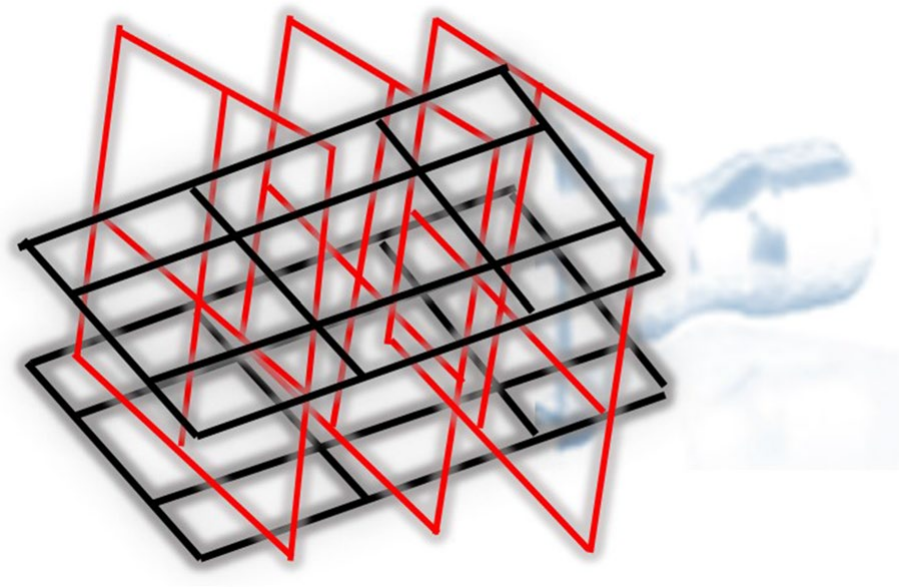
Interpenetrating network structure of hybrid composite resin

Hybrid ceramics are another example of biomimetic ceramic materials. They were introduced in a trial to combine the advantages of ceramics and composites in order to get physical properties (e.g., Youngs modulus and hardness) similar to that of enamel and dentin [32]. Polymers infiltrated ceramic network (PICN) materials consist of two interlocking phases, a porous feldspathic ceramic network (75–80% of the volume), infiltrated with polymer [33]. Recently, researchers of material science have developed functionally graded PICN that are characterized by gradients in composition and structure to produce a structure that have properties of enamel and dentine combined (Fig. 5) [1].

Since the tooth is made up of different structures (enamel and dentin) which have different optical and physical properties, the possibility of mimicking both tissues with one layer of material is not an easy task. Recently manufacturers began to implement the concept of gradient shade and translucency in their ceramic products. Furthermore, the concept of biomimetics was recently extended to include gradients in strength within the same ceramic block. A novel multilayered zirconia block that integrates different types of zirconia together in one block was introduced. These new blocks are monolithic restorations that contains yttria partially stabilized tetragonal zirconia polycrystal (3Y-TZP) in the body area to provide high mechanical stability and 5Y-TZP in the occlusal area to provide the desired esthetics [34].

## Biomimetic regenerative dentistry

### Dentine remineralisation

Demineralisation is the process of removing mineral ions from Hydroxyapatite (HA) crystals of enamel, dentin, cementum, and bone. Depositing lost mineral ions again to the demineralized crystals is called remineralization. Considerable number of mineral ions could be lost from HA without destroying its integrity but high sensitivity to heat, cold, pressure, and pain would be expected which is termed dentin hypersensitivity. Once the integrity of HA crystal lattice becomes lost, dental cavities can be clearly seen. Demineralisation is not an irreversible process; hence, the demineralized HA crystals can grow back to their original size under favorable remineralisation conditions [35, 36].

- Crystallite’s growth conventional remineralisation approach

**Fig. 6:**
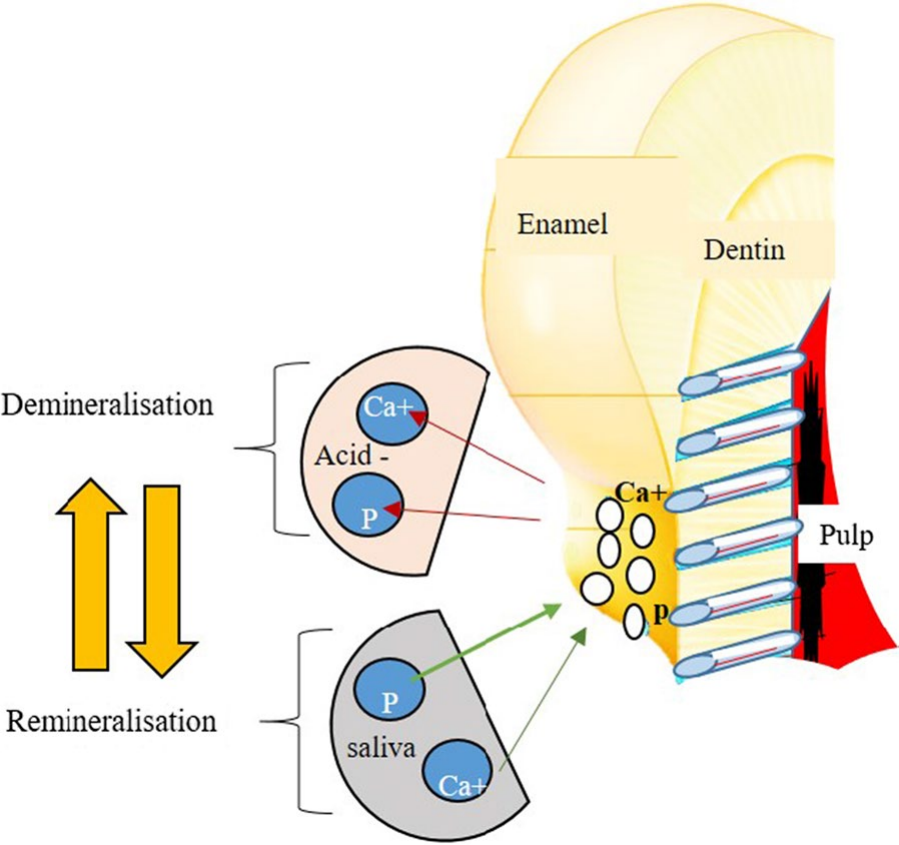
Demineralisation and remineralisation processes

Conventional remineralisation protocols of the carious dentin often involve using formulations with calcium and phosphate ions of different concentrations. In this case, remineralisation occurs by epitaxial growth of residual apatite crystals in partially demineralized dentin rather than new crystals nucleation [35, 36]. If there are no or very few residual crystals, there will be no remineralisation. The mineral content of the surface layer of the lesion affects the quality of the resulting remineralisation, including its location and mineral deposition density (Fig. 6) [37].

b. Bottom-up biomimetic remineralisation approaches

**Fig. 7:**
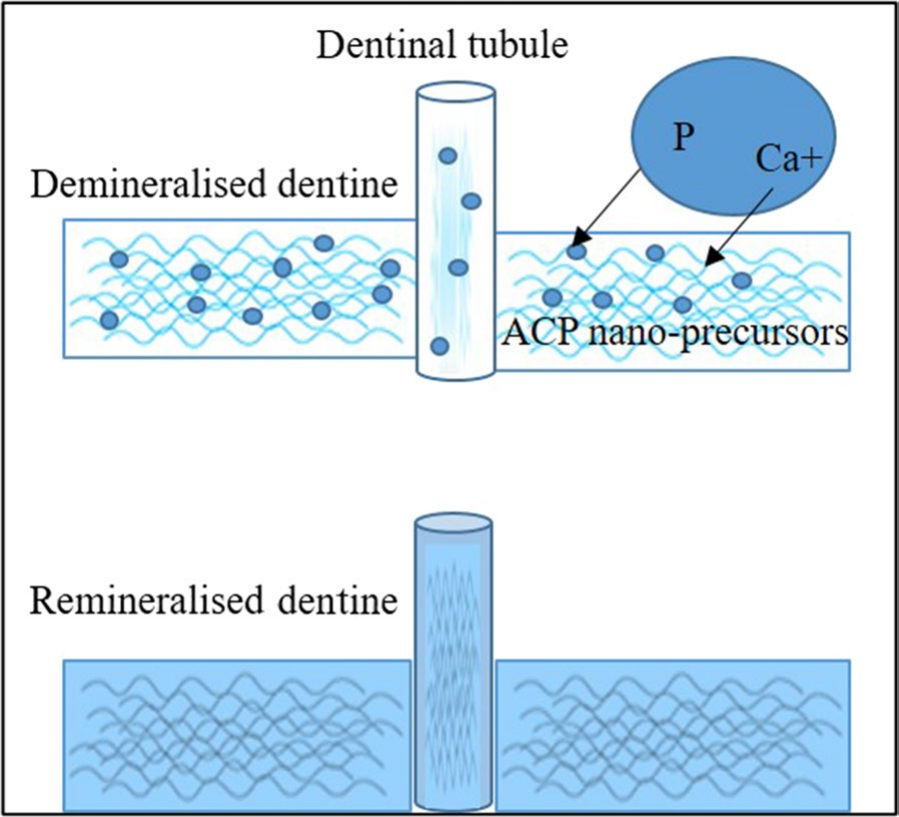
Bottom up biomimetic approach

The biomimetic remineralisation process is a bottom-up approach used to create nanocrystals that are small enough to fit in the gaps between adjacent collagen molecules in order to backfill the demineralized dentin collagen. These nano precursor particles (amorphous calcium phosphate (ACP) nano-precursors) are stabilized by biomimetic analogs of non-collagenous proteins (dentin matrix protein (DMP1) and dentin phosphophoryn (DPP, DMP2) that regulate the HA crystal nucleation growth [38, 39]. In this direction, several bioactive materials and non-collagenous proteins (NCPs) analogs have been used to promote remineralisation (Fig. 7) [40].

### Biomimetic dentin remineralisation materials

#### Bioactive materials

- Bioactive glass (BAG)

In 1969, Hench developed the first bioactive glass (BAG) [41]. BAG has showed a remarkable regenerative capacity for repairing hard tissues due to the ability to release ions for apatite crystals formation that mimics the natural apatite found in bone and dental hard tissues [42].

BAG is characterized by its unique composition that made it a prospective bioactive material for several medical uses. It is a mixture of ingredients that are common constituents of the natural human hard tissues (sodium, calcium, phosphorous and silica) [43]. In dentistry, BAG is used in tissue engineering and for coating titanium and ceramic implants to promote bone regeneration [44].

When BAG contacts water or saliva, it releases calcium, sodium, and phosphorous ions, which promotes remineralisation at surfaces of dental tissues. Moreover, the calcium phosphate precipitates occlude the dentinal tubules and prevent movement of dentinal fluids, which is responsible for dentine hypersensitivity according to the hydrodynamic theory [45]. BAG can also be used in pulp capping procedures since they are non-cytotoxic and were reported to promote the formation of reparative dentin [44].

b. Casein phosphopeptide–amorphous calcium phosphate (CPP-ACP)

CPP-ACP is a bioactive complex that is formulated from casein phosphopeptide (CPP), a milk product derived from casein protein and amorphous calcium phosphate (ACP), a precursor in the biological formation of hydroxyapatite. CPP-ACP has substantial potential to prevent enamel demineralisation and enhance enamel and dentine remineralisation [46]. CPP-ACP contained toothpastes, sugar-free chewing gums, mouth rinses, and biomaterials (composite resin, glass ionomer, etc.) showed efficacy in triggering hydroxyapatite precipitation and tissue repair [47–49].

CPP-ACP mechanism of action relies on the ability of CPP to maintain high concentrations of calcium and phosphorus ions and stabilize them in the form of ACP to avoid spontaneous crystallization. Furthermore, CPP-ACP nano-complex localize the calcium, phosphate, and hydroxide ions at the tooth surface and maintain a state of super saturation in the plaque fluid [50]. When the pH drops (≤ 5.8), hydroxyapatite starts to precipitate and infiltrate into the subsurface to achieve efficient enamel remineralisation at the lesion area. Moreover, the combination of CPP-ACP and fluoride was reported to boost the remineralisation effect of carious and erosive lesions [51].

c. Fluoride compounds

Remineralisation is considered a natural repair process that involves the reformation of hydroxyapatite below the surface of non-cavitated lesions from calcium and phosphate ions found in the saliva [52]. In the presence of fluoride ions, fluoroapatite crystals are formed instead of HA. The critical pH of fluoroapatite is lower than that of hydroxyapatite which makes it more acid resistant. Furthermore, the presence of fluoride in the plaque fluid and in contact with tooth surface was found to protect the hydroxyapatite crystals against dissolution and promote remineralisation by adhering to calcium and phosphorus ions [53].

Fluoride can be incorporated in toothpastes or applied directly to the tooth surface through fluoride varnish as prophylaxis [53]. Direct application of fluoride compounds allows fluoride to be maintained intra-orally for a longer time and is slowly released into the saliva forming a fluoride reservoir for extended protection effect. Very recently, a patented technology named REFIX with a biomimetic mechanism of action was developed. It is a toothpaste containing fluoride in combination with phosphates and silica. Such combination favors the formation of a fluoridated apatite and a silicon-enriched layer which incorporate deep into the hydroxyapatite and occlude the dentinal tubule openings [54].

Biomimetic mineralisation (BIMIN) is another technique developed to stimulate guided formation of a layer of fluorapatite that resembles enamel. This is done on a mineral substrate and has the potential to enhance the remineralisation of superficial enamel and exposed demineralised dentin. This technique based on diffusion of calcium ions from a solution into a glycerin-enriched gel with phosphate and fluoride ions. When the conditioned gel is in direct contact with the exposed tooth surface, a mineral layer that is firmly adherent to the tooth’s surface is formed within eight hours [55].

d. Zinc oxide loaded materials

Zinc has been widely used in dentistry and experimentally added into several dental materials, not only to reduce MMPs (matrix metalloproteinases)-mediated collagen degradation, but also to induce dentin remineralisation [56]. Zinc is considered a bioactive element improving the repair ability of demineralised dentin. It stimulates protein phosphorylation, enhances calcium deposition and facilitates dentinal tubule occlusion by crystals precipitation. Moreover, these precipitated crystals are resistant to dissolution in acidic environment, thus offer stable results [57].

#### Non-collagenous proteins (NCPs) analogues

- Polydopamine

Polydopamine (PDA) is a semi conductive polymer that is formed by the oxidation and polymerization of dopamine in aqueous solutions [58]. It is called biological glue due to its strong ability to bond to several substrates under wet conditions [59].

The adhesive property of polydopamine is inspired from mussels which depend on a secreted 3,4-dihydroxy-L-phenylalanine (DOPA) enriched protein that allows it to adhere to surfaces under water [59]. PDA was reported to effectively promote enamel and dentin remineralisation simultaneously. It binds to Ca^2+^ ions and acts as nucleation site for the formation of biomimetic hydroxyapatite in the presence of saliva [60]. PDA can be deposited in very thin films (≤ 100 nm) on almost all kinds of material surfaces. Because PDA coatings are rich in active catechol and amine functional groups, they can be used as bonding agents or a biomimetic coating for various restorative materials [61].

b. Polyelectrolytes

Polyelectrolytes are polymers consisting mainly of macromolecules that contain ionic groups which dissociate in aqueous solutions and become charged [62]. Polyvinylphosphonic acid (PVPA) is one of the polyelectrolytes that are used in dentistry. Its biomimetic property is related to its ability to form nanocrystals that stimulate intrafibrillar and interfibrillar remineralisation at the resin-dentin interface. PVPA prevent hybrid layer degradation during dentin bonding since it acts as a protease inhibitor of MMPs in dentine, which are responsible for endogenous degradation of dentin collagen fibrils [63]. Furthermore, PVPA has been used as a biomimetic analog for phosphoproteins like dentin matrix acidic phosphoprotein 1 (DMP1) and dentin phosphoprotein (DPP).DMP 1 and DPP are extracellular matrix proteins, which are very crucial for bone and dentin remineralisation [64].

Polyacrylic acid (PAA) is another polyelectrolyte that is also used as the NCP analogues in biomimetic mineralization of dentine. PAA simulates the calcium phosphate binding sites of DMP1. Moreover, PAA can stabilize metastable ACP nano-precursors that are small enough to infiltrate the demineralised collagen matrix [65, 66].

c. Polyamidoamine Dendrimer (PAMAM)

Dendrimers are called artificial proteins due to their biomimetic abilities and extensive application in medicine. Dendrimers are spherical and branched structure in shape with many surface groups. They promote biomimetic intrafibrillar mineralization of dentin by following structural hierarchy. PAMAM has the ability to absorb calcium and phosphate ions via its functional groups and thus stimulate and activate enamel and dentin remineralisation [67]. They are considered potent remineralising materials as they can cause sequestration of the mineral ions and can act as template for nucleation. Poly (amidoamine) is a notable dendrimer in bio mineralisation fields, because it performs in the same manner as non-collagenous proteins (NCPs) [68, 69]**.**

d. Sodium trimetaphosphate (STMP)

STMP (Na_3_P_3_O_9_) is a phosphophoryn analog that has the potential to phosphorylate collagen type I and produce negatively charged sites that attract nano precursors. The use of STMP is considered a biomimetic innovative strategy to stabilize and strengthen the dentin through interaction with non-collagenolytic proteins, mineral nucleation and remineralisation, and decreasing collagen biodegradation.

### Biomimetic dentine-pulp complex regeneration

Carious lesions when left untreated will extend beyond dentin and cause inflammation and infection of the pulp and may reach the periapical tissues as well [70]. Treatment strategies in this situation are categorized into vital and non-vital pulp therapies. The aim of vital pulp therapy is to stimulate dentine bridge formation in order to protect and isolate the pulp tissue. For inflamed pulp or non-vital pulp tissue, until today, root canal treatment is the standard care process. It involves debridement and cleaning of the tooth canals from all diseased pulp tissues and sealing the canals with an inert material to prevent any micro leakage [70].

The conventional treatment of a necrotic pulp is problematic due to increased risk of root fracture, pain, and secondary infections [71]. Biomimetic regeneration of the dentin-pulp complex is a contemporary approach that involves disinfecting the root canal system followed by applying bioactive material such as biomimetic cement, growth factors, stem cells or scaffolds. These materials and approaches have the ability to control signaling and differentiation of the pulp cells to limit inflammatory responses and to allow tissue repair and regeneration [72, 73]. This should also promote continued deposition of new dentin-like hard tissue. More research is still needed to exactly assess whether and how this newly deposited dentin-like hard tissue strengthens the pre-existing weak root [72, 73].

#### Bioactive materials

Bioactivity of dental materials refers to their ability to induce local physiological responses to form a chemical bond or tissue formation through chemical or physical interaction. Remineralizing bioactive materials have the ability to precipitate surface appetite-like material when immersed in physiological body fluid, remineralization stimulation through adding minerals to tooth structure besides promotion of normal tissue repair [74].

- Calcium hydroxide (Ca OH_2_)

Ca (OH)_2_ is a white odorless strong alkaline powder (pH > 12) that slowly dissolve in water. It was first introduced to dentistry in 1920 by Herman as direct pulp capping material. In aqueous environment, calcium hydroxide slowly releases Ca_2_^+^ and OH^−^ ions, which are responsible for its antibacterial and hard tissue regeneration effects. The released hydroxyl ions attack the bacterial cell and cause damage to the bacterial DNA and cytoplasmic membrane [75].

Furthermore, the hydroxyl group raises the pH and activates the alkaline phosphatase enzymes, which encourage repair and calcifications of dentin through the deposition of calcium phosphate in the dentin organic matrix. Moreover, the alkaline pH of Ca (OH)_2_ neutralizes the acidic effect of lactic acid released from osteoclasts, thereby preventing demineralisation of dentin. Direct contact of Ca (OH)_2_ with pulp connective tissue creates a necrotic zone rich in calcium salts and calcium-protein complexes and stimulates the formation of reparative dentin bridge [75].

Calcium hydroxide is involved in many dental applications such as pulp capping, pulpotomy, apexification and root canal disinfection as well as in treatment of root resorption, root or furcation perforations, and horizontal tooth fracture. Even though calcium hydroxide has some challenges, such as poor sealing ability and rapid degradation, it is still a material of choice for various endodontic therapies owing to its ease of handling and economic cost compared to other materials like mineral trioxide aggregate (MTA).

b. Mineral Trioxide Aggregate

Mineral trioxide aggregate (MTA) is a calcium silicate based hydrophilic cement that was developed by Torabinejad in 1993 as a modification of Portland cement [76]. MTA is composed mainly of dicalcium silicate, tricalcium silicate and tricalcium aluminate in addition to radio-opacifiers to make it suitable for clinical use [77]. Like other calcium silicate-based cements, MTA is biocompatible, bioactive and exhibit positive odontogenic effect on dental pulp cells and peripheral root tissues [73].

The bioactivity of MTA has been attributed to ability of calcium ions to react with phosphate in the presence of phosphate-buffered saline yielding hydroxyapatite layer at the MTA-dentin interface [78]. MTA is advocated for several dental applications such as pulp capping, retrograde filling, apexification, and management of furcation and root perforations [78].

As compared to calcium hydroxide, MTA is more stable and has greater sealing ability, no moisture sensitivity, better handling, and more favorable outcomes in maintaining long-term pulp vitality [79]. Mineral Trioxide Aggregate material with very high plasticity (MTA Repair HP) has been recently launched, representing a calcium silicate-based cement with enhanced physical and mechanical properties. It is highly biocompatible with human pulp stem cells and with a proven bio-mineralization and antimicrobial activity [80, 81].

c. Biodentine

The first commercially available biodentine was introduced in 2009 as a dentine-substitute material. Biodentine is a bioactive calcium-silicate based material that penetrates through opened dentinal tubules and interlocks with dentin to boost the mechanical properties. The main powder components of biodentine are tricalcium silicate and dicalcium silicate [82]. Other minor constituents include calcium carbonate, iron oxide, and zirconium oxide. The liquid consists of calcium chloride, which acts as accelerator, and a hydrosoluble polymer [83].

It is used for pulp capping material, root perforation, apexification stimulant, and as a retrograde filling material. Biodentine induces odontoblast differentiation of human Dental Pulp Stem Cells (DPSCs) causing faster mineralization of pulp tissue due to the release of transforming growth factor (TGF-Beta 1) [84, 85]. It has advantages over MTA in terms of ease of handling, short setting time, high viscosity, and strength [82]. Biodentine is characterized by having similar compressive strength as natural dentine [86]. The compressive strength is considered critical for pulp therapy products as they should be able to withstand external impacts without leakage [87]. Another advantage of biodentin is its ability to establish high bond strength to different adhesive systems [88]. This is of particular importance when used as a dentine substitute material under adhesive bonded restorations.

d. Enamel Matrix Derivative (EMD)

Enamel Matrix Derivative refers to a purified protein derived from the enamel layer of a developing porcine tooth germ cell [89]. EMD consists mainly of amelogenins (90%) and smaller amounts of enamelin, ameloblastin, amelotin and other proteins. EMD was reported to play a major role in regeneration of the periodontal ligament. The mechanism of action of EMD is still not fully explained, but it is believed that application of amelogenins onto a conditioned root surface forms an extracellular matrix with high affinity for hydroxyapatite and collagen, which interacts with the surrounding cells and thus initiates regeneration [90]. EMD has been also proved to induce reparative dentin in direct pulp capping and stimulating odontogenesis. In addition, other uses were reported such as preventing implantitis, replantation cases, sealing of root perforations, pulpotomy and as intracanal medication [91, 92].

#### Cell homing strategies

Cell homing strategies for dental tissue regeneration was introduced in 2010, representing a viable approach for tissue regeneration [93]. It involves recruitment of stem or progenitor cells in order to achieve tissue regeneration and revascularization by chemotaxis via biological signalling molecules [94].

- Platelet concentrates

Platelet concentrates are a group of autologous biomaterials that are naturally present in the human body and play significant role in tissue regeneration and wound healing [95]. They are derived from peripheral blood and offer a series of advantages including ease of accessibility, biocompatibility, low cost, and regenerative potentiality. The platelet concentrates are composed of biological regenerative material, mainly platelets and fibrin [96].

Platelet-rich plasma (PRP) was first described in the seventies of the last century as a source of growth factors such as platelet-derived growth factor (PDGF), transforming growth factor-β (TGF-β) and insulin-like growth factor-1 (IGF-1) [96]. The final extract is obtained through double centrifugation process. PRP is commonly used in various surgical procedures such as ridge augmentation, cleft palate repair, sinus lifting, soft tissue grafts etc. owing to its attribute to accelerate soft tissue healing [95].

**Fig. 8:**
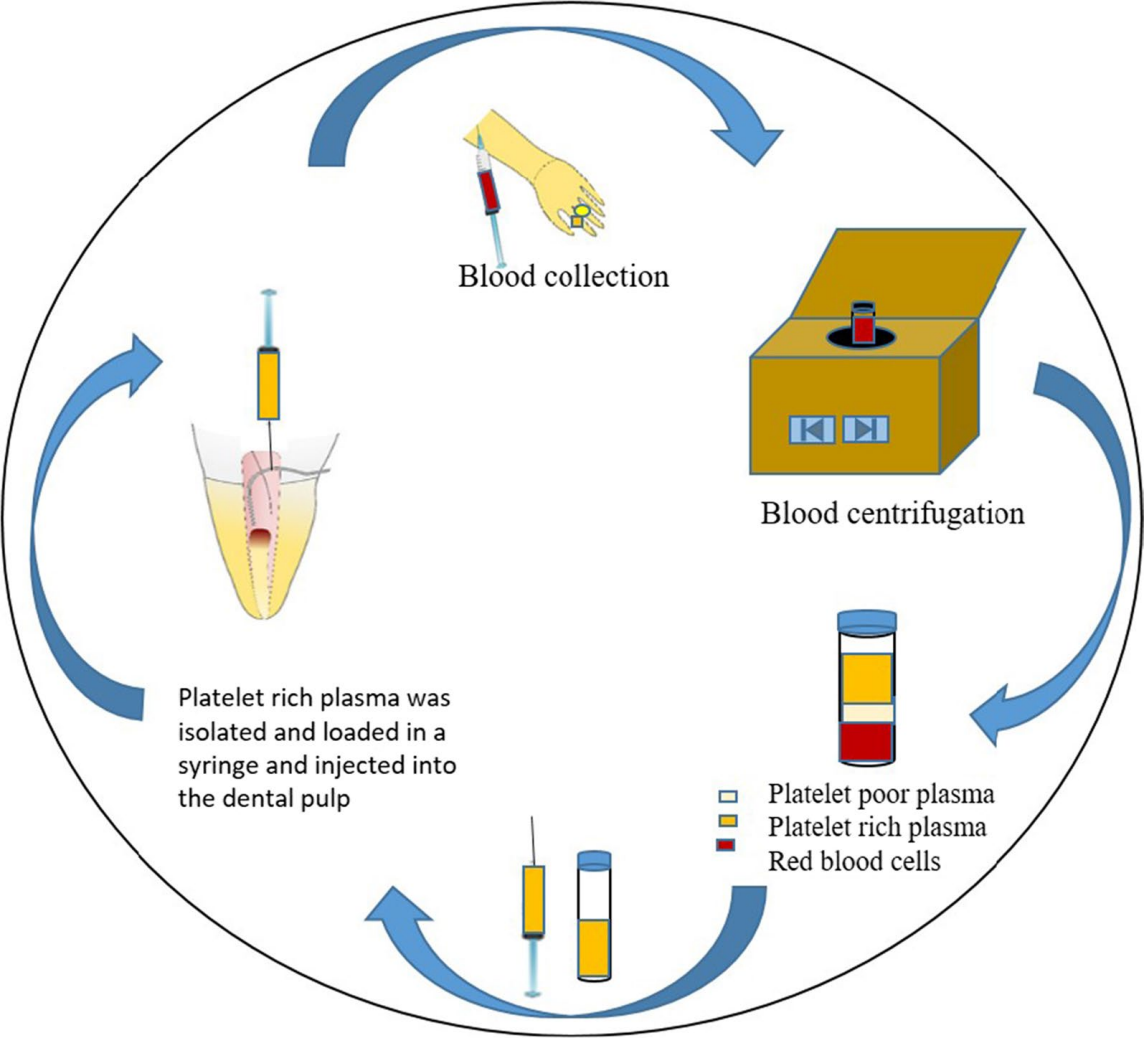
Platelet concentrates extraction process

Furthermore, PRP is an ideal scaffold for regenerative endodontic treatment regimens because of its ability to maintain the vitality of pulp tissues by promoting cell growth and transport of growth factors in a disinfected environment. Platelet-rich fibrin (PRF) was developed as a second-generation platelet concentrate. It has several advantages over PRP such as simple and cost-effective method of preparation, and no need for addition of any exogenous anticoagulant compounds [97]. PRF contains biologically active proteins such as cytokines, gluconic chains, and structural glycoproteins, which are entangled within fibrin network [97]. This biological complex has been known to boost rapid wound healing and trigger periodontal regeneration (Fig. 8) [95].

b. Bone morphogenetic proteins

Bone morphogenetic proteins (BMPs) are dimeric molecules, which belong to the transforming growth factor ß (TGF-ß) superfamily and consists of two polypeptide chains connected by single disulfide bond [98]. BMPs are found in demineralized bone matrices and dentin and are also primary key players in embryonic tooth development and cytodifferentiation. BMP4 and BMP5 are involved in the process of ameloblast differentiation, whereas BMP2, BMP4, MBP6 and BMP7 stimulate the differentiation of odontoblast [99].

Scientists were able to use recombinant human BMP2 to induce the differentiation of pulp cells into odontoblasts. Other recombinant BMPs were able to stimulate the formation of reparative dentin. BMP-induced dentin regeneration could be stimulated by direct application of BMPs into pulp tissue to endorse natural healing. Another approach is external differentiation of isolated progenitor stem cells and subsequently transplanting the differentiated odontoblast into the tooth [100]. Carriers or transmitters play significant role to facilitate the clinical application of BMPs and ensure bio-accessibility and gradual distribution of BMPs to the host tissues. They must be biodegradable and biocompatible. Calcium hydrate and calcium phosphate were reported to be used as carriers for BMPs [101].

c. Hyaluronic acid (HA) and derivatives

HA is one of the major basic glycosaminoglycan components of the dental pulp extracellular matrix (ECM). HA can interact with stem cell receptors to induce regeneration, play a role in dentin matrix, and pulp tissue development. Moreover, HA derivatives induces mineralization and odontogenic differentiation. Although HA is biocompatible, biodegradable, and bioactive, it has low mechanical properties and requires growth factors to improve its regenerative potentials [70, 102].

d. Collagen

A natural biocompatible bioactive material that mimics ECM. It provides bioactivity by facilitating adhesion and attachment of stem cells, and induces signaling pathways that promote differentiation [103].

## Conclusions

Biomimetic dentistry could open a new era through successful repair and replacement of diseased dental hard and soft tissues. Restorative dentistry in the future would no longer be using inert materials that only fill the prepared cavity, but instead, it will rely mainly on bioactive materials with the capability of dental tissues regeneration. Biomimetic mineralization of dentine with different methods, including the use of NCP analogues and biomimetic materials showed promising results for demineralized carious dentine. New mechanisms for tissue engineering and regeneration of the dentin pulp complex using biomimetic technologies and concepts can emerge as a major turnover in the dental field.

## Data Availability

All data generated or analysed during this study are included in this published article.

## Abbreviations

GIC: Glass-ionomer cement
DCR: Dental composite resin
CAD/CAM: Computer aided design/computer assisted manufacturing
PICN: Polymers infiltrated ceramic network
3Y-TZP: 3-Mol % yttria partially-stabilized tetragonal zirconia polycrystal
HA: Hydroxyapatite
PAPE: Phosphoric acid polyethylene
MMPs: Matrix metalloproteinases
PAA: Polyacrylic acid
PAMAM: Polyamidoamine dendrimer
STMP: Sodium trimetaphosphate
CPP-ACP: Casein phosphopeptide amorphous calcium phosphate
BAG: Bioactive glass
ACP: Amorphous calcium phosphate
NCPs: Non-collagenous proteins
DMP: Dentin matrix protein
Ca (OH)_2_: Calcium hydroxide
MTA: Mineral trioxide aggregate
PDA: Polydopamine
PVPA: Polyvinyl phosphonic acid
PRP: Platelet-rich plasma
PDGF: Platelet-derived growth factor
TGF-β: Transforming growth factor-β
IGF-1: Insulin-like growth factor-1
BMPs: Bone morphogenetic proteins
ECM: Extracellular matrix
HA: Hyaluronic acid

## References

[1] Eldafrawy M, Nguyen JF, Mainjot AK, Sadoun MJ (2018). A functionally graded PICN material for biomimetic CAD-CAM blocks. J Dent Res.

[2] Goswami S (2018). Biomimetic dentistry. J Oral Res Rev.

[3] Hwang J, Jeong Y, Park JM, Lee KH, Hong JW, Choi J (2015). Biomimetics: forecasting the future of science, engineering, and medicine. Int J Nanomedicine.

[4] Fayemi PE, Wanieck K, Zollfrank C, Maranzana N, Aoussat A (2017). Biomimetics: process, tools and practice. Bioinspir Biomim.

[5] Cramer MD (1997). Biomimicry: innovation inspired by nature—Benyus. JM Libr J.

[6] Bazos P, Magne P (2011). Bio-emulation: biomimetically emulating nature utilizing a histo-anatomic approach; structural analysis. Eur J Esthet Dent.

[7] Slavkin HC (1996). Biomimetics: replacing body parts is no longer science fiction. J Am Dent Assoc.

[8] Zafar MS, Amin F, Fareed MA, Ghabbani H, Riaz S, Khurshid Z, Kumar N (2020). Biomimetic aspects of restorative dentistry biomaterials. Biomimetics (Basel).

[9] Burke FJ (2003). From extension for prevention to prevention of extension: (minimal intervention dentistry). Dent Update.

[10] Malhotra S, Hegde M (2015). Analysis of marginal seal of ProRoot MTA, MTA Angelus biodentine, and glass ionomer cement as root-end filling materials: An in vitro study. J Oral Res Rev.

[11] Furtos G, Cosma V, Prejmerean C, Moldovan M, Brie M, Colceriu A, Vezsenyi L, Silaghi-Dumitrescu L, Sirbu C (2005). Fluoride release from dental resin composites. Mater Sci Eng C-Biomim Supramol Syst.

[12] Nicholson JW, Croll TP (1997). Glass-ionomer cements in restorative dentistry. Quintessence Int.

[13] Alatawi RAS, Elsayed NH, Mohamed WS (2019). Influence of hydroxyapatite nanoparticles on the properties of glass ionomer cement. J Mater Res Technol.

[14] Garoushi S, Vallittu P, Lassila L (2017). Hollow glass fibers in reinforcing glass ionomer cements. Dent Mater.

[15] Singer L, Bierbaum G, Kehl K, Bourauel C (2020). Evaluation of the antimicrobial activity and compressive strength of a dental cement modified using plant extract mixture. J Mater Sci Mater Med.

[16] Katiyar NK, Goel G, Hawi S, Goel S (2021). Nature-inspired materials: emerging trends and prospects. Npg Asia Materials.

[17] Ravi RK, Alla RK, Shammas M, Devarhubli A (2013). Dental composites-A versatile restorative material: an overview. Indian J Dent Sci.

[18] Yeli M, Kidiyoor K, Nain B, Kumar P (2010). Recent advances in composite resins-a review. J Oral Res Rev.

[19] Fugolin APP, Pfeifer CS (2017). New resins for dental composites. J Dent Res.

[20] Diesendruck CE, Sottos NR, Moore JS, White SR (2015). Biomimetic self-healing. Angew Chem Int Ed Engl.

[21] Trask RS, Williams HR, Bond IP (2007). Self-healing polymer composites: mimicking nature to enhance performance. Bioinspir Biomim.

[22] Wertzberger BE, Steere JT, Pfeifer RM, Nensel MA, Latta MA, Gross SM (2010). Physical characterization of a self-healing dental restorative material. J Appl Polym Sci.

[23] Bijelic-Donova J, Keulemans F, Vallittu PK, Lassila LVJ (2020). Direct bilayered biomimetic composite restoration: the effect of a cusp-supporting short fiber-reinforced base design on the chewing fracture resistance and failure mode of molars with or without endodontic treatment. J Mech Behav Biomed.

[24] Lassila L, Sailynoja E, Prinssi R, Vallittu PK, Garoushi S (2020). Bilayered composite restoration: the effect of layer thickness on fracture behavior. Biomater Investig Dent.

[25] Magne P (2006). Composite resins and bonded porcelain: The postamalgam era?. J Calif Dent Assoc.

[26] Cattell MJ, Chadwick TC, Knowles JC, Clarke RL, Samarawickrama DY (2006). The nucleation and crystallization of fine grained leucite glass-ceramics for dental applications. Dent Mater.

[27] Fouda AM, Atta O, Kassem AS, Desoky M, Bourauel C (2022). Wear behavior and abrasiveness of monolithic CAD/CAM ceramics after simulated mastication. Clin Oral Investig.

[28] Holand W, Rheinberger V, Wegner S, Frank M (2000). Needle-like apatite-leucite glass-ceramic as a base material for the veneering of metal restorations in dentistry. J Mater Sci Mater Med.

[29] Goudouri OM, Kontonasaki E, Papadopoulou L, Kantiranis N, Lazaridis NK, Chrissafis K, Chatzistavrou X, Koidis P, Paraskevopoulos KM (2014). Towards the synthesis of an experimental bioactive dental ceramic. Part I: crystallinity characterization and bioactive behavior evaluation. Mater Chem Phys.

[30] Goudouri OM, Kontonasaki E, Papadopoulou L, Manda M, Kavouras P, Triantafyllidis KS, Stefanidou M, Koidis P, Paraskevopoulos KM (2017). An experimental bioactive dental ceramic for metal-ceramic restorations: textural characteristics and investigation of the mechanical properties. J Mech Behav Biomed.

[31] Ferraris M, Verne E, Appendino P, Moisescu C, Krajewski A, Ravaglioli A, Piancastelli A (2000). Coatings on zirconia for medical applications. Biomaterials.

[32] Dirxen C, Blunck U, Preissner S (2013). Clinical performance of a new biomimetic double network material. Open Dent J.

[33] Albero A, Pascual A, Camps I, Grau-Benitez M (2015). Comparative characterization of a novel cad-cam polymer-infiltrated-ceramic-network. J Clin Exp Dent.

[34] Michailova M, Elsayed A, Fabel G, Edelhoff D, Zylla IM, Stawarczyk B (2020). Comparison between novel strength-gradient and color-gradient multilayered zirconia using conventional and high-speed sintering. J Mech Behav Biomed.

[35] Sauro S, Osorio R, Osorio E, Watson TF, Toledano M (2013). Novel light-curable materials containing experimental bioactive micro-fillers remineralise mineral-depleted bonded-dentine interfaces. J Biomater Sci Polym Ed.

[36] Shen C, Zhang NZ, Anusavice KJ (2010). Fluoride and chlorhexidine release from filled resins. J Dent Res.

[37] Nancollas GH, Wu WJ (2000). Biomineralization mechanisms: a kinetics and interfacial energy approach. J Cryst Growth.

[38] Niu LN, Zhang W, Pashley DH, Breschi L, Mao J, Chen JH, Tay FR (2014). Biomimetic remineralization of dentin. Dent Mater.

[39] Tay FR, Pashley DH (2008). Guided tissue remineralisation of partially demineralised human dentine. Biomaterials.

[40] Liu Y, Mai S, Li N, Yiu CK, Mao J, Pashley DH, Tay FR (2011). Differences between top-down and bottom-up approaches in mineralizing thick, partially demineralized collagen scaffolds. Acta Biomater.

[41] Hench LL (2006). The story of bioglass. J Mater Sci Mater Med.

[42] Hench LL (2013). Chronology of bioactive glass development and clinical applications. New J Glass Ceram.

[43] van Gestel NA, Geurts J, Hulsen DJ, van Rietbergen B, Hofmann S, Arts JJ (2015). Clinical applications of S53P4 bioactive glass in bone healing and osteomyelitic treatment: a literature review. Biomed Res Int.

[44] Baino F, Hamzehlou S, Kargozar S (2018). Bioactive glasses: Where are we and where are we going?. J Funct Biomater.

[45] Fernando D, Attik N, Pradelle-Plasse N, Jackson P, Grosgogeat B, Colon P (2017). Bioactive glass for dentin remineralization: a systematic review. Mater Sci Eng C Mater Biol Appl.

[46] Reema SD, Lahiri PK, Roy SS (2014). Review of casein phosphopeptides-amorphous calcium phosphate. Chin J Dent Res.

[47] Marovic D, Sariri K, Demoli N, Ristic M, Hiller KA, Skrtic D, Rosentritt M, Schmalz G, Tarle Z (2016). Remineralizing amorphous calcium phosphate based composite resins: the influence of inert fillers on monomer conversion, polymerization shrinkage, and microhardness. Croat Med J.

[48] Mazzaoui SA, Burrow MF, Tyas MJ, Dashper SG, Eakins D, Reynolds EC (2003). Incorporation of casein phosphopeptide-amorphous calcium phosphate into a glass-ionomer cement. J Dent Res.

[49] Yassin O, Milly H (2019). Effect of CPP-ACP on efficacy and postoperative sensitivity associated with at-home vital tooth bleaching using 20% carbamide peroxide. Clin Oral Investig.

[50] Bayram M, Kusgoz A, Yesilyurt C, Nur M (2017). Effects of casein phosphopeptide-amorphous calcium phosphate application after interproximal stripping on enamel surface: an in-vivo study. Am J Orthod Dentofacial Orthop.

[51] Wang CP, Huang SB, Liu Y, Li JY, Yu HY (2014). The CPP-ACP relieved enamel erosion from a carbonated soft beverage: an in vitro AFM and XRD study. Arch Oral Biol.

[52] Farooq I, Moheet IA, AlShwaimi E (2015). In vitro dentin tubule occlusion and remineralization competence of various toothpastes. Arch Oral Biol.

[53] Cardoso Cde A, Lacerda B, Mangueira DF, Charone S, Olympio KP, Magalhaes AC, Pessan JP, Vilhena FV, Sampaio FC, Buzalaf MA (2015). Mechanisms of action of fluoridated acidic liquid dentifrices against dental caries. Arch Oral Biol.

[54] Vilhena FV, de Oliveira SML, Matochek MHM, Tomaz PLS, Oliveira TS, D'Alpino PHP (2021). Biomimetic mechanism of action of fluoridated toothpaste containing proprietary REFIX technology on the remineralization and repair of demineralized dental tissues: an in vitro study. Eur J Dent.

[55] Guentsch A, Seidler K, Nietzsche S, Hefti AF, Preshaw PM, Watts DC, Jandt KD, Sigusch BW (2012). Biomimetic mineralization: long-term observations in patients with dentin sensitivity. Dent Mater.

[56] Osorio R, Osorio E, Cabello I, Toledano M (2014). Zinc induces apatite and scholzite formation during dentin remineralization. Caries Res.

[57] Toledano M, Vallecillo-Rivas M, Aguilera FS, Osorio MT, Osorio E, Osorio R (2021). Polymeric zinc-doped nanoparticles for high performance in restorative dentistry. J Dent.

[58] Wu H, Zhao C, Lin K, Wang X (2022). Mussel-inspired polydopamine-based multilayered coatings for enhanced bone formation. Front Bioeng Biotechnol.

[59] Liu C, Liu J, Ning X, Chen S, Liu Z, Jiang S, Miao D (2019). The effect of polydopamine on an Ag-coated polypropylene nonwoven fabric. Polymers (Basel).

[60] Zhang J, He X, Yu S, Zhu J, Wang H, Tian Z, Zhu S, Cui Z (2021). A novel dental adhesive containing Ag/polydopamine-modified HA fillers with both antibacterial and mineralization properties. J Dent.

[61] Zhou YZ, Cao Y, Liu W, Chu CH, Li QL (2012). Polydopamine-induced tooth remineralization. ACS Appl Mater Interfaces.

[62] Lu ZQ, Zhang LL, Yan YK, Wang W (2021). Polyelectrolytes of inorganic polyoxometalates: acids, salts, and complexes. Macromolecules.

[63] Xie Y, He E, Cao Z, Ou Q, Wang Y (2019). Effect of polyvinylphosphonic acid on resin-dentin bonds and the cytotoxicity of mouse dental papilla cell-23. J Prosthet Dent.

[64] Louis H, Berman KMH (2020). Cohen's pathways of the pulp.

[65] Cao CY, Mei ML, Li QL, Lo EC, Chu CH (2015). Methods for biomimetic remineralization of human dentine: a systematic review. Int J Mol Sci.

[66] Gu L, Kim YK, Liu Y, Ryou H, Wimmer CE, Dai L, Arola DD, Looney SW, Pashley DH, Tay FR (2011). Biomimetic analogs for collagen biomineralization. J Dent Res.

[67] Liang K, Wang S, Tao S, Xiao S, Zhou H, Wang P, Cheng L, Zhou X, Weir MD, Oates TW, Li J, Xu HHK (2019). Dental remineralization via poly(amido amine) and restorative materials containing calcium phosphate nanoparticles. Int J Oral Sci.

[68] Bae J, Son WS, Yoo KH, Yoon SY, Bae MK, Lee DJ, Ko CC, Choi YK, Kim YI (2019). Effects of poly(amidoamine) dendrimer-coated mesoporous bioactive glass nanoparticles on dentin remineralization. Nanomaterials (Basel).

[69] Tao SY, Fan ML, Xu HHK, Li JS, He LB, Zhou XD, Liang KN, Li JY (2017). The remineralization effectiveness of PAMAM dendrimer with different terminal groups on demineralized dentin in vitro. RSC Adv.

[70] Upadhyay A, Pillai S, Khayambashi P, Sabri H, Lee KT, Tarar M, Zhou S, Harb I, Tran SD (2020). Biomimetic aspects of oral and dentofacial regeneration. Biomimetics (Basel).

[71] Landys Boren D, Jonasson P, Kvist T (2015). Long-term survival of endodontically treated teeth at a public dental specialist clinic. J Endod.

[72] Miller EK, Lee JY, Tawil PZ, Teixeira FB, Vann WF (2012). Emerging therapies for the management of traumatized immature permanent incisors. Pediatr Dent.

[73] Tawil PZ, Duggan DJ, Galicia JC (2015). Mineral trioxide aggregate (MTA): its history, composition, and clinical applications. Compend Contin Educ Dent.

[74] Hamdy T (2018). Bioactivity: a new buzz in dental materials. EC Dent Sci.

[75] Nishanthi R, Ravindran V (2020). Role of calcium hydroxide in dentistry: a review. Int J Pharm Res.

[76] Prati C, Gandolfi MG (2015). Calcium silicate bioactive cements: biological perspectives and clinical applications. Dent Mater.

[77] Altan H, Tosun G (2016). The setting mechanism of mineral trioxide aggregate. J Istanb Univ Fac Dent.

[78] Cervino G, Laino L, D'Amico C, Russo D, Nucci L, Amoroso G, Gorassini F, Tepedino M, Terranova A, Gambino D, Mastroieni R, Tozum MD, Fiorillo L (2020). Mineral trioxide aggregate applications in endodontics: a review. Eur J Dent.

[79] Mostafa NM, Moussa SA (2018). Mineral trioxide aggregate (MTA) vs calcium hydroxide in direct pulp capping–literature review. On J Dent Oral Health.

[80] Abu Hasna A, de Paula RL, Campos TMB, de Castro Lopes SLP, Rachi MA, de Oliveira LD, Carvalho CAT (2022). Biological and chemical properties of five mineral oxides and of mineral trioxide aggregate repair high plasticity: an in vitro study. Sci Rep.

[81] Cintra LTA, Benetti F, de Azevedo Queiroz IO, de Araujo Lopes JM, Penha de Oliveira SH, Sivieri Araujo G, Gomes-Filho JE (2017). Cytotoxicity, biocompatibility, and biomineralization of the new high-plasticity MTA material. J Endod.

[82] Kaur M, Singh H, Dhillon JS, Batra M, Saini M (2017). MTA versus biodentine: review of literature with a comparative analysis. J Clin Diagn Res.

[83] Malkondu O, Karapinar Kazandag M, Kazazoglu E (2014). A review on biodentine, a contemporary dentine replacement and repair material. Biomed Res Int.

[84] Laurent P, Camps J, About I (2012). Biodentine(TM) induces TGF-beta1 release from human pulp cells and early dental pulp mineralization. Int Endod J.

[85] Luo Z, Kohli MR, Yu Q, Kim S, Qu T, He WX (2014). Biodentine induces human dental pulp stem cell differentiation through mitogen-activated protein kinase and calcium-/calmodulin-dependent protein kinase II pathways. J Endod.

[86] O'Brien WJ (2008). Dental materials and their selection.

[87] Hashem DF, Foxton R, Manoharan A, Watson TF, Banerjee A (2014). The physical characteristics of resin composite-calcium silicate interface as part of a layered/laminate adhesive restoration. Dent Mater.

[88] Odabaş ME, Bani M, Tirali RE. Shear bond strengths of different adhesive systems to biodentine. Sci World J 2013;2013: 626103 . 10.1155/2013/626103 PMC3809944 24222742

[89] Miron RJ, Sculean A, Cochran DL, Froum S, Zucchelli G, Nemcovsky C, Donos N, Lyngstadaas SP, Deschner J, Dard M, Stavropoulos A, Zhang Y, Trombelli L, Kasaj A, Shirakata Y, Cortellini P, Tonetti M, Rasperini G, Jepsen S, Bosshardt DD (2016). Twenty years of enamel matrix derivative: the past, the present and the future. J Clin Periodontol.

[90] Alberti A, Francetti L, Taschieri S, Corbella S (2021). The applications of enamel matrix derivative in implant dentistry: a narrative review. Materials (Basel).

[91] da Silva KTL, Grazziotin-Soares R, de Miranda RR, Novais VR, Carvalho EM, da Silva GR, Bauer J, Carvalho CN (2022). Effect of an enamel matrix derivative (Emdogain) on the microhardness and chemical composition of human root dentin: an in vitro study. Sci Rep.

[92] Riksen EA, Landin MA, Reppe S, Nakamura Y, Lyngstadaas SP, Reseland JE (2014). Enamel matrix derivative promote primary human pulp cell differentiation and mineralization. Int J Mol Sci.

[93] Kim JY, Xin X, Moioli EK, Chung J, Lee CH, Chen M, Fu SY, Koch PD, Mao JJ (2010). Regeneration of dental-pulp-like tissue by chemotaxis-induced cell homing. Tissue Eng Part A.

[94] Kumar N, Maher N, Amin F, Ghabbani H, Zafar MS, Rodriguez-Lozano FJ, Onate-Sanchez RE (2022). Biomimetic approaches in clinical endodontics. Biomimetics (Basel).

[95] Saini K, Chopra P, Sheokand V (2020). Journey of platelet concentrates: a review. Biomed Pharmacol J.

[96] Xu J, Gou L, Zhang P, Li H, Qiu S (2020). Platelet-rich plasma and regenerative dentistry. Aust Dent J.

[97] Dohan DM, Choukroun J, Diss A, Dohan SL, Dohan AJ, Mouhyi J, Gogly B (2006). Platelet-rich fibrin (PRF): a second-generation platelet concentrate. Part I: technological concepts and evolution. Oral Surg Oral Med Oral Pathol Oral Radiol Endod.

[98] Granjeiro JM, Oliveira RC, Bustos-Valenzuela JC, Sogayar MC, Taga R (2005). Bone morphogenetic proteins: from structure to clinical use. Braz J Med Biol Res.

[99] Díaz-Sánchez R-M, Yáñez-Vico R-M, Fernández-Olavarría A, Mosquera-Pérez R, Iglesias-Linares A, Torres-Lagares D (2015). Current approaches of bone morphogenetic proteins in dentistry. J Oral Implantol.

[100] Liang C, Liang Q, Xu X, Liu X, Gao X, Li M, Yang J, Xing X, Huang H, Tang Q, Liao L, Tian W (2022). Bone morphogenetic protein 7 mediates stem cells migration and angiogenesis: therapeutic potential for endogenous pulp regeneration. Int J Oral Sci.

[101] Huang K-H, Wang C-Y, Chen C-Y, Hsu T-T, Lin C-P (2021). Incorporation of calcium sulfate dihydrate into a mesoporous calcium silicate/poly-ε-caprolactone scaffold to regulate the release of bone morphogenetic protein-2 and accelerate bone regeneration. Biomedicines.

[102] Chrepa V, Pitcher B, Henry MA, Diogenes A (2017). Survival of the apical papilla and its resident stem cells in a case of advanced pulpal necrosis and apical periodontitis. J Endod.

[103] Gong T, Heng BC, Lo EC, Zhang C (2016). Current advance and future prospects of tissue engineering approach to dentin/pulp regenerative therapy. Stem Cells Int.

